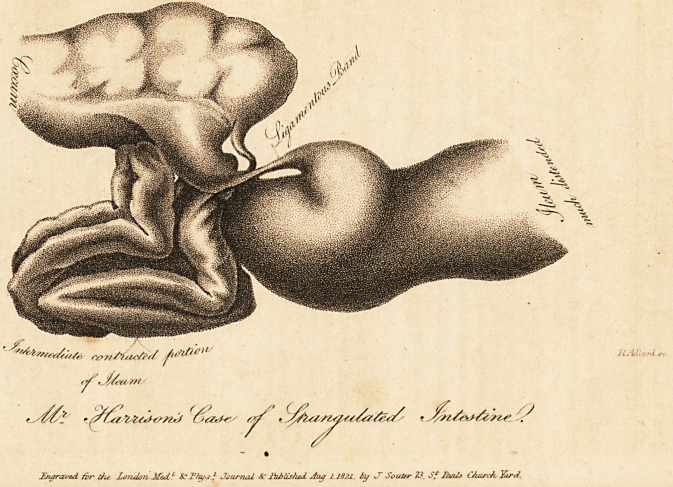# Account of a Case of Obstruction of the Intestinal Canal

**Published:** 1821-08

**Authors:** John Harrison

**Affiliations:** Member of the Royal College of Surgeons, and Assistant Surgeon 1st or Grenadier Regiment of Guards.


### Original Communications, Select Observations, etc.

---

*Account of a Case of Obstruction of the Intestinal Canal.* By JOHN HARRISON, Member of the Royal College of Surgeons, and Assistant Surgeon 1st or Grenadier Regiment of Guards.

CASE of Corporal William Brandige, aged 30.—Date of admission, March 7, 1819.—The subject of this curious and fatal case of obstruction of the intestinal canal, was a soldier. His symptoms, on admission into the hospital on the evening of the 7th of March, were—pain and griping in the bowels, inclination to vomit; has had no stool the three preceding days; pulse small and frequent; skin not above the natural temperature; tongue clean. Ordered to bed; was bled, and had a purge given.

8*th.*—No effect from the purge; the abdomen has become tense and somewhat painful to the touch, accompanied with an accession of febrile action. He was again more largely bled; the purging medicines were repeated. Ordered the warm bath, and to continue in it until some degree of faintness should come on. In the afternoon, as no stool had yet been procured, an enema was thrown up, but was soon returned, without bringing away the least feculent matter.

9*th.*—He experienced much relief this morning, by vomiting a very large quantity of fluid feculent matter. The stomach now rejected almost every thing it received. A tobacco-glyster was thrown up to-day, and induced faintness and much debility. Purging medicines, in the form of pills, were retained, but produced no effect, and every means adopted to procure a passage proved ineffectual.

10*th.*—Symptoms accompanying the latter stage of strangulated hernia were now coming on, and seemed to preclude every hope of relief. The poor fellow's sufferings were great indeed: our attention became henceforth more particularly directed to palliate them. He always found some abatement by vomiting up this feculent matter, which he continued to do more or less till his death, and this was protracted till the thirtieth day after his admission; thus having had no passage through him for three-and-thirty days. It seems surprising that he could have lingered this length of time, considering the alarming symptoms which were observed on the 10th.

On examination after death, the small intestines were found immensely distended with flatus, and in a state of gangrene: they seemed to have acquired an unusually large size, and, when laid open, the valvulæ conniventes were most beautifully exhibited. This distended appearance ended abruptly within about two feet before the ileum terminates in the cœcum, which space of ileum was quite contracted and empty, but appeared healthy. The cause of all this mischief was a firm ligamentous band, (as seen in the engraving,) two inches in length, connecting two points of ileum together, the contracted portion being the intermediate space: this band proceeded at the superior part, from a pouch. The obstruction appeared to have been caused by the ligamentous band passing firmly across that part of the gut immediately below the pouch, and binding it down so as absolutely to obstruct the passage; or we may, perhaps, more properly say, that this part of the gut was placed accidentally, or became entangled in this situation, beneath the band, from a distended state of the superior part.

The man's general appearance, when living, had always been healthy, and I could not learn that he had at any time been particularly subject to bowel complaints. The band was a perfect *lusus naturæ*, and not a connexion or adhesion from previous inflammation.

## Figures and Tables

**Figure f1:**